# PI3K pathway activation results in low efficacy of both trastuzumab and lapatinib

**DOI:** 10.1186/1471-2407-11-248

**Published:** 2011-06-15

**Authors:** Leiping Wang, Qunling Zhang, Jian Zhang, Si Sun, Haiyi Guo, Zhen Jia, Biyun Wang, Zhimin Shao, Zhonghua Wang, Xichun Hu

**Affiliations:** 1 Department of Medical Oncology, Fudan University Shanghai Cancer Center; Department of Oncology, Shanghai Medical College, Fudan University, Shanghai 200032, China; 2 Department of Breast Surgery, Fudan University Shanghai Cancer Center; Department of Oncology, Shanghai Medical College, Fudan University, Shanghai 200032, China

## Abstract

**Background:**

Human epidermal growth factor receptor 2 (HER2) is the most crucial ErbB receptor tyrosine kinase (RTK) family member in HER2-positive (refered to HER2-overexpressing) breast cancer which are dependent on or "addictive" to the Phosphatidylinositol-3-kinase (PI3K) pathway. HER2-related target drugs trastuzumab and lapatinib have been the foundation of treatment of HER2--positive breast cancer. This study was designed to explore the relationship between PI3K pathway activation and the sensitivity to lapatinib in HER2--positive metastatic breast cancer patients pretreated with anthracyclins, taxanes and trastuzumab.

**Methods:**

Sixty-seven HER2-positive metastatic breast cancer patients were recruited into a global lapatinib Expanded Access Program and 57 patients have primary tumor specimens available for determination of PI3K pathway status. PTEN status was determined by immunohistochemical staining and PIK3CA mutations were detected via PCR sequencing. All patients were treated with lapatinib 1250 mg/day continuously and capecitabine 1000 mg/m^2 ^twice daily on a 2-week-on and 1-week-off schedule until disease progression, death, withdrawal of informed consent, or intolerable toxicity.

**Results:**

PIK3CA mutations and PTEN loss were detected in 12.3% (7/57) and 31.6% (18/57) of the patients, respectively. Twenty-two patients with PI3K pathway activation (defined as PIK3CA mutation and/or PTEN expression loss) had a lower clinical benefit rate (36.4% versus 68.6%, P = 0.017) and a lower overall response rate (9.1% versus 31.4%, P = 0.05), when compared with the 35 patients with no activation. A retrospective analysis of first trastuzumab-containing regimen treatment data showed that PI3K pathway activation correlated with a shorter median progression-free survival (4.5 versus 9.0 months, P = 0.013).

**Conclusions:**

PIK3CA mutations occur more frequently in elder patients for HER2-positive breast cancer. PIK3CA mutations and PTEN loss are not mutually exclusive. PI3K pathway activation resulting from PTEN loss or PIK3CA mutations may lead to drug resistance to lapatinib and trastuzumab (http://ClinicalTrials.gov number, NCT00338247).

## Background

Human epidermal growth factor receptor 2 (HER2) is the most crucial ErbB receptor tyrosine kinase (RTK) family member in breast cancer with overexpression in about one fourth of patients [1]. Since HER2 plays a key role in HER2-positive breast cancer, these patients usually have bad prognosis, and HER2-related target drugs have been the foundation of treatment. Trastuzumab, a HER2 monoclonal antibody against the extracellular domain of the molecule, has been a new standard in neo-adjuvant, adjuvant and palliative treatment of HER2-positive breast cancer [1-3]. However, trastuzumab mono-therapy shows a response rate of no more than 30% in palliative setting [4], and there is still a problem of primary or acquired resistance even with combination regimens. HER2-overexpressing breast cancer cells are dependent on or "addictive" to the Phosphatidylinositol-3-kinase (PI3K) pathway [5]. Published literatures showed that PI3K pathway activation is associated with primary resistance to trastuzumab, and trastuzumab exerts its antitumor effects only in the presence of a normal PI3K pathway [6-11].

PI3K pathway is one of the most important signaling pathways in cell, which is involved in many fundamental cellular processes, including proliferation, cell survival, motility and cell growth [12,13]. Class IA PI3K, the most important member of the PI3K complex, is composed of a heterodimer with a p85 regulatory subunit and a p110 catalytic subunit (PIK3CA), residing downstream of multiple receptor kinase families including ErbB RTK family (EGFR, HER2, HER3, HER4) and transducing signals originating from them [12,13]. Phosphatase and tensin homolog deleted on chromosome 10 (PTEN) is a phosphotase that converts membrane-associated phosphatidylinositol 3,4,5-triphosphate (PIP3) back to phosphatidylinositol 4,5-bisphosphate (PIP2) and negatively regulates signaling transduction of PI3K pathway [14,15]. It is well known that dysregulation of PI3K pathway plays an important role in the development of malignancy, and the most common genetic alterations in this pathway are PIK3CA mutation and PTEN loss [16,17], both of which can lead to constitutive activation of PI3K pathway and resistance to trastuzumab [7]. PTEN-related resistance to trastuzumab can be reversed by combined treatment with trastuzumab and the PI3K inhibitor LY294002 [18]. Therefore, PI3K pathway activation resulting from PIK3CA mutation and/or PTEN loss warrants further studies.

Up to now, little knowledge is available about the correlation between PI3K pathway status and efficacy and resistance of the other FDA-approved anti-HER2 agent, lapatinib. Laptinib, a dual tyrosine kinase inhibitor of EGFR and HER2, binds to the intracellular kinase domain [19]. It has no cross-resistance with trastuzumab since it is effective against breast cancer expressing p95HER2 [20], an active truncated form of HER2 and with HER2 epitope masked by mucin 4 [21]. Clinical data have shown the safety and efficacy of lapatinib alone and in combination with capecitabine, paclitaxel and letrozole and it is still effective in patients who have progressed on trastuzumab [22-24]. Therefore, the HER2 pathway is still an "addictive" oncogenic pathway in breast cancer pretreated with trastuzumab. However, several recent papers touching on PI3K pathway activation and lapatinib resistance conflicted with each other [18,25-29], so we conducted this study to explore their correlation and the protocol was approved by the Fudan University Shanghai Cancer Center Institutional review board on June 30, 2008.

## Methods

### Patient Eligibility and Study Design

A global lapatinib Expanded Access Program was started to offer preapproval drug in order to provide clinical benefit to patients with HER2-positive metastatic breast cancer who had progressive diseases on treatment with regimens including anthracyclines, taxanes, and trastuzumab. Trastuzumab had to be used in metastatic setting. Tumors with either 3+ immunohistochemical staining for HER2 protein or HER2 gene amplification by fluorescence in situ hybridization were defined as HER2 positive in our institution. Women previously treated with capecitabine were eligible. Patients were required to have evaluable disease according to the Response Evaluation Criteria in Solid Tumors (RECIST); an Eastern Cooperative Oncology Group (ECOG) performance status of 0 or 1; a left ventricular ejection fraction (LVEF) within the institution's normal range; a life expectancy of at least 12 weeks; and adequate renal, hepatic, and hematologic function. patients with central nervous system (CNS) metastases were eligible if they were clinically stable for at least 3 months after discontinuation of radiation therapy. patients with preexisting heart disease or conditions that could affect gastrointestinal absorption were ineligible. All patients gave written informed consent on recruitment into the global lapatinib Expanded Access Program and provision of the primary tumor sample for this translational study(http://ClinicalTrials.gov number, NCT00338247).

In this one-arm study, all patients receive the combination regimen consisting of lapatinib at a dose of 1250 mg daily on a continuous basis and capecitabine at a dose of 2000 mg per square meter of body-surface area in two divided doses on days 1 through 14 of a 21-day cycle. Standard recommendations for capecitabine dosage modifications were followed in the management of adverse events. Lapatinib was withheld for up to 14 days for grade 2 or more nonhematologic toxicity or any grade 3 or 4 hematologic toxicity. Patients were assessed every 6 weeks for the first 24 weeks, and then every 12 weeks while they were still receiving the study treatment. Patients who had no progressive disease but whose study treatment was withdrawn were assessed every 12 weeks until the commencement of alternative anticancer treatment, disease progression, or death. Efficacy was determined according to the RECIST criteria. Adverse events were assessed according to the National Cancer Institute's Common Terminology Criteria for Adverse Events (CTCAE, version 3.0). The clinical benefit was defined as a complete response, partial response, or stable disease for at least 6 months. Progression-free survival was calculated as the interval between the date of signing informed consent and the date of disease progression, or death from any cause.

### PCR Sequencing and PIK3CA Mutation

DNA was extracted from formaldehyde-fixed, paraffin-embedded tumor tissue. PCR were performed with 10 to 100 ng of genomic DNA as template following a standard protocol. PIK3CA gene PCR primers were E9F CAAAGCAATTTCTACACGAGATCC; E9R GTAAAAACATGCTGAGATCAGCCACAT; E20F TGGAATGCCAGAACTACAATCTTT; E20R GGTCTTTGCCTGCTGAGAGTT. The PCR products were sequenced using the ABI3130XL automated capillary sequencer by Shanghai Tianhao Biotechnology Company.

### Immunohistochemistry and PTEN Scoring

The antibody for PTEN IHC staining was a rabbit monoclonal anti-PTEN (D4.3) XPTM (Cell Signaling Beverly, MA, USA) diluted 1:200. The antibody was applied overnight at 4ºC. Then the tissues were incubated with the second antibody (1:500, Jackson lab) for 30 min. The color was developed with DAB solution about 1 min at room temperature and then stained in Harris hematoxylin solution for 3 min. Each set of slides included positive and negative control slides and normal cells in a tumor were used as an internal control.

PTEN immunoreactivity was examined by two independent observers who were blinded to the clinical data. A third pathologist was invited when the discordance was present between them. The staining was mainly visible in the cytoplasm of tumor cells. PTEN expression levels were semiquantified using immunoreactive scores (IRS) calculated by multiplying the percentage of PTEN-positive tumor cells (scored 0 to 4) with the PTEN staining intensity [1-3]. The tumor was graded as PTEN-negative (IRS 0-3), weak positive (IRS 4-6), positive (IRS 7-9), and strong positive (IRS 10-12)[6].

### Statistical Analysis

The relationships between different variables were assessed by Chi-square tests and the trends were also examined by Chi-square tests when required. Differences in progression-free survival (PFS) and overall survival (OS) between groups were determined using the log-rank test. After a univariate analysis, the variables with significant correlation with PFS and OS, continuous variables and PI3K pathway status were put in a Cox proportional hazard regression model to determine which was an independent prognostic factor for PFS and OS, respectively. The statistical difference was considered significant if the P value was less than 0.05.

## Results

### Patient Characteristics

Sixty-seven Chinese patients were enrolled from Fudan University Cancer Center from Aug. 2008 to Mar. 2010. The median age was 49.0 years old (range 26-75). Fifty-seven patients had their tumor tissues available for detection of PI3K pathway activation (PIK3CA mutation and PTEN expression loss). Clinical and pathological data for the patients are showed in Table 1.

**Table 1:** Patient Characteristics

Characteristics	All	Patients	Patients	analyzed	P value
	**No**.	Percent	**No**.	Percent	
Age, (yrs)					
Median(IQR)	49.0 (46.5-51.4)		49.5 (46.8-52.3)		P > 0.05
Range	26-75		26-75		
EOCG performance status					
0	12	17.9%	10	17.5%	P > 0.05
1	49	73.1%	45	79.0%	
2	6	9.0%	2	3.5%	
Hormone receptor status					
ER(+) or PR(+) or Both	29	43.3%	25	43.8%	P > 0.05
ER(-) and PR(-)	38	56.7%	32	56.2%	
HER2 expression by IHC					
1+/2+ (HER2 status confirmed by FISH)	22	32.8%	20	35.1%	P > 0.05
3+ positive	45	67.2%	37	64.9%	
Number of involved sites					
1	16	23.9%	13	22.8%	P > 0.05
2	23	34.3%	22	38.6%	
≥3	28	41.8%	22	38.6%	
Location of involved sites					
Visceral only	19	28.4%	16	28.1%	P > 0.05
Visceral and nonvisceral	34	50.7%	29	50.9%	
Nonvisceral only	14	20.9%	12	21.0%	
Involved organs					
Lung	29	43.3%	26	45.6%	P > 0.05
Liver	26	38.8%	23	40.4%	
Brain	18	26.9%	14	24.6%	
Previous chemotherapy regimens					
1	16	23.9%	13	22.8%	P > 0.05
2	22	32.8%	20	35.1%	
≥3	29	43.3%	24	42.1%	
Previous capecitabine					
YES					
Progression during capecitabine	35	52.2%	29	50.9%	P > 0.05
No Progression during capecitabine	11	16.4%	10	17.5%	
NO	21	31.3%	18	31.6%	

Legend: ER, estrogen receptor; PR, progesterone receptor; IHC, immunohistochemistry; FISH, fluorescence in situ hybridization

### PIK3CA mutation and PTEN expression loss

**Figure 1:**
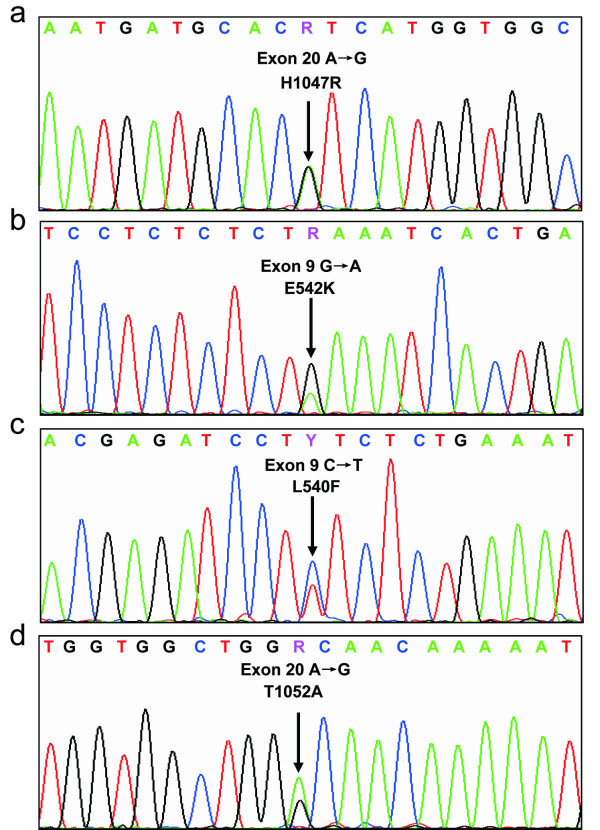
**PTEN gene mutations**. Sample 38, 46, 65, 68 have exon 20 missense mutations, H1047R (a); sample 2 has exon 9 missense mutation, E542K (b); sample 11 has exon 9 missense mutation, L540F (c); sample 54 has exon 20 missense mutation, T1052A (d).

The overall incidence of PIK3CA mutations was 12.3% (7 in 57 samples). The majority of mutations occurred at two hotspots, H1047R (7%, 4 samples) at exon 20 encoding the kinase domain (Figure 1a), and E542K (1.8%, 1 sample) at exon 9 encoding the helical domain (Figure 1b). L540F and T1052A mutations are rare and each was found in one tumor sample (Figure 1c-d).

**Figure 2:**
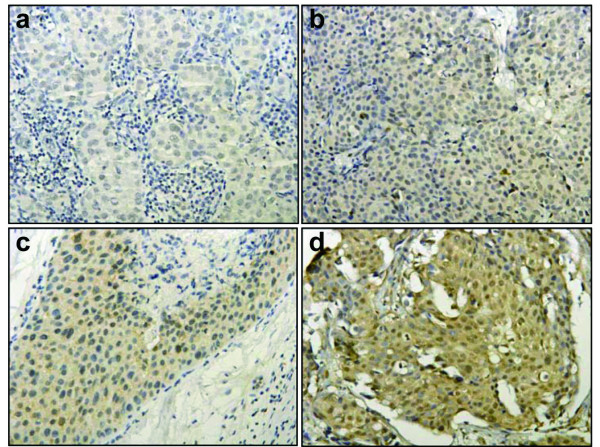
**PTEN expression**. PTEN negative (IRS 0-3, a); Weak Positive (IRS 4-6, b); Positive (IRS 7-9, c); and Strong Positive (IRS 10-12, d).

PTEN expression loss was found in 18 patients (31.6%, Figure 2a). Thirty-nine patients were positive for PTEN expression [6], in which 17 (29.8%), 14 (24.6%), and 8 (14%) specimens were weak positive, positive and strong positive respectively (Figure 2b-d). In this study, PTEN loss was not mutually exclusive with PIK3CA mutations, since 3 of the 4 patients with H1047R mutation were also found to have no PTEN expression (Table 2).

**Table 2:** Summary of PI3KCA mutations and PTEN expression

		PI3KCA	mutations		Wild Type	Total
			
PTEN expression	Exon	20	Exon	9		
(IHC score)	H1047R	T1052A	E542K	L540F		
negative	3	0	0	0	15	18
weak positive	1	0	0	0	16	17
positive	0	1	1	0	12	14
strong positive	0	0	0	1	7	8
Total	4	1	1	1		

Legend: IHC, immunohistochemistry

Compared with the wild type, PI3K pathway activation (PIK3CA mutation and/or PTEN expression loss) was identified in a significantly older patient population (P = 0.016). The median age of patients with the PI3K pathway activation was 53.6 ± 7.9 years, while the median age of those with no PI3K pathway activation was 47.0 ± 10.9 years. The PI3K pathway activation status was not associated with all other clinicopathological parameters, such as hormone receptor status, HER2 protein expression status and disease free interval after radical mastectomy (all P > 0.05).

### Patient outcome and PI3K pathway activation

On September 30, 2010, preliminary analysis was made on the basis of 50 disease progression events and 28 deaths. Median follow-up time was 15.3 ± 6.3 months. The median PFS of all 67 patients was 6.5 months (95%CI, 5.7-7.3 months), and the median PFS of the 57 patients who provided their tumor tissues for detection of PI3K pathway activation was also 6.5 months (95%CI, 5.4-7.6 months). The overall response rate (complete response plus partial response) was 22.4% for all 67 patients and 22.8% for the 57 patients whose tumor sample were available. The corresponding clinical benefit rates were 58.2% and 56.1%. The median overall survivals for both cohorts were 17.0 months.

**Figure 3:**
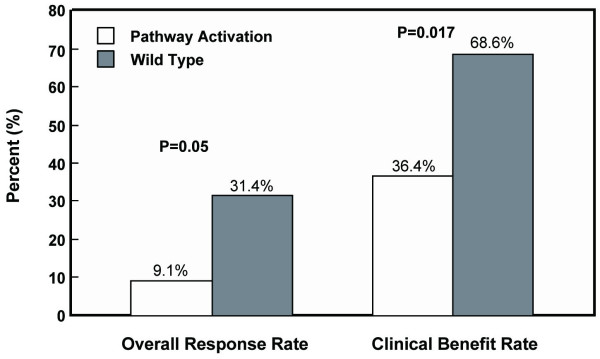
**Overall response rate (ORR) and clinical benefit rate (CBR) grouped by the status of PI3K pathway activation**. The ORR was marginally significant between pathway activation group and wild type group; the CBR was statistically significant between pathway activation and wild type groups.

**Figure 4:**
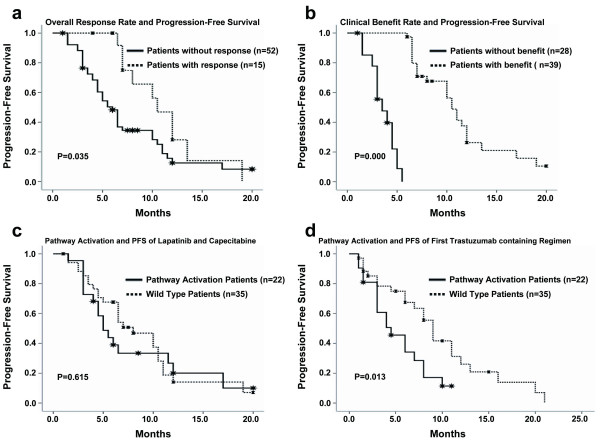
**Progression-free survival with objective response, clinical benefit, and PI3K pathway activation**. The difference in PFS between patients with response (CR+PR) and those without response (a); the difference in PFS between patients with clinical benefit (CR+PR+SD > 6 months) and those without benefit (b); the difference in PFS between patients with pathway activation and those with no activation (c), and pathway activation status with efficacy of the first Trastuzumab-containing regimen (d).

An analysis of our data showed that PIK3CA mutation didn't correlate with overall response rate, clinical benefit rate or progression-free survival. For PTEN expression status, patients with wild type gene enjoyed a clinical benefit rate of 66.7%, which was statistically higher than 33.3% in those with no PTEN expression (P = 0.018). The overall response rate of 28.2% and median PFS of 8 months in the patients with PTEN expression were substantially higher, although the differences were not statistically significant (Table 3). When analyzing PIK3CA mutation together with PTEN expression loss since both can activate PI3K pathway, the clinical benefit was still observed for patients with no activation of PI3K pathway (68.6% versus 36.4%, P = 0.017; Figure 3). The overall response rate was also higher (31.4% versus 9.1%, P = 0.05; Figure 3). Both overall response and clinical benefit significantly correlated with PFS (Figure 4a-b), however, there was no significant association of PI3K pathway activation status with PFS or OS (Figure 4c).

**Table 3:** PI3K pathway activation and Efficacy of HER2-Targeted Drugs

	PI3KCA mutation			PTEN expression			PI3K pathway activation		
	
	Mutation (n = 7)	WT (n = 50)	P	Loss (n = 18)	WT (n = 39)	P	Activation (n = 22)	WT (n = 35)	P
Lapatinib plus Capecitabine									
Overall response No.(%)	1 (14.3%)	12 (24%)	NS	2 (11.1%)	11 (28.2%)	NS	2 (9.1%)	11 (31.4%)	0.050
Clinical benefit No. (%)	3 (42.9%)	29 (58%)	NS	6 (33.3%)	26 (66.7%)	0.018	8 (36.4%)	24 (68.6%)	0.017
Median PFS months (95% CI)	6 (2.7-9.3)	6.5 (4.3-8.7)	NS	5 (4.0-6.0)	8 (5.6-10.4)	NS	5 (3.5-6.6)	8 (5.1-10.9)	NS
Median OS months (95% CI)	17 (2.5-31.5)	17 (11.8-22.2)	NS	19.5	15	NS	19.5	15	NS
First Trastuzumab Regimen									
Median PFS months (95% CI)	4.5 (0.7-8.3)	8 (5.2-10.8)	NS	6 (3.3-8.7)	9 (7.1-10.9)	0.024	4.5 (2.0-7.0)	9 (7.6-10.4)	0.013

Legend: NS, not significant; WT, wildtype gene

A retrospective analysis was done to explore the relationship between PI3K pathway activation status and the efficacy of the other anti-HER2 drug, trastuzumab. We chose the progression-free survival of the first trastuzumab-containing regimen as an indicator for trastuzumab efficacy. The regimens were trastuzumab combined with one or two chemotherapy drugs, including docetaxel, vinorelbine, paclitaxel, gemcitabine, capecitabine and cisplatin. As previously reported, PI3K pathway activation shortened the median progression-free survival significantly (4.5 versus 9.0 months, P = 0.013; Figure 4d). PTEN expression status had a similar differentiating effect (6.0 versus 9.0 months, P = 0.024). However, the difference of PFS (4.5 versus 8.0 months) resulting from PIK3CA mutation wasn't significant.

### Patient outcome and other factors

Response and survival of breast cancer might be affected by many other factors, such as age, ECOG performance status, hormone receptor status, HER2 expression, metastatic sites, number of metastatic sites and previous treatments. A univariate analysis of our data showed that only number of metastatic sites had a negative impact on overall response rate of the lapatinib and capecitbine doublet, while none of the above-mentioned factors was statistically associated with clinical benefit rate. Concerning survival, only previous capecitabine treatment was statistically associated with a shorter PFS (6.5 vs. 11.0 months, P = 0.011, Table 4) and OS (14.0 months vs. not reached, P = 0.003).

**Table 4:** Summary of Patients outcome and other factors

Characteristics	Lapatinib plus capecitabine Median PFS (months)	P value
Age, (yrs)		

<49 yr	6	0.283
≥49 yr	10	
EOCG performance status		
0	10	0.174
1 or 2	6.5	
Hormone receptor status		
ER(+) or PR(+) or Both	6.5	0.529
ER(-) and PR(-)	7	
HER2 expression by IHC		
1+/2+ (HER2 status confirmed by FISH)	6	0.963
3+ positive	7	
Number of involved sites		
1 or 2	8.5	0.249
≥3	6.5	
Location of involved sites		
Visceral only	6.5	0.544
Visceral and nonvisceral	7.0	
Nonvisceral only	7.0	
Previous chemotherapy regimens		
1 or 2	10	0.078
≥3	6.5	
Previous capecitabine		
YES	6.5	0.011
NO	11	

We put all the above-mentioned variants of PI3K pathway status, age and clinical benefit status into Cox's proportional hazards model to investigate how much PI3K pathway activation affected patient outcome. Clinical benefit rate was the only independent factor for PFS in HER2-positive breast cancer.

## Discussion

PI3K pathway activation is the most common signal transduction pathway alteration in malignancies, including breast cancer [30]. It mostly results from PIK3CA mutation or amplification, PTEN loss [16]. Since the patients enrolled in this study were HER2-positive and high-level PIK3CA amplification without mutation is rare in this subpopulation [31,32], so only PIK3CA mutation and PTEN loss were determined for the samples in our study.

Our data showed that PIK3CA mutation occurred in 12.3% of the patients, lower than 8-40% reported in other studies [33-38]. There are several reasons. First, only two exons accounting for approximately 85% of all mutations were determined in our study [33]. Second, mutation correlates with an old age and this phenomenon was confirmed by our study [39]. However, the median age of our patients was 49.0 years, about 10 years younger than Caucasian counterparts [39]. Third, the mutation was reported to occur more frequently in HER2-negative patients [39,40], however, all patients in our study were HER2-positive.

Regarding mutations in hot spots, two common mutation points, H1047R and E542K were also present in our patients with no mutation of E545K observed [37,39]. As to mutations in non-hot spots, two new points, L540F and T1052A mutations were first reported based on our knowledge. An analysis of our data showed that the ratio of hot spots to non-hot spots was 2.5 to 1, which is consistent with other reports [39]. Since there were only a few patients with the new mutation, our result needed further confirmation by other studies. Therefore, it remains a question whether the new mutation in non-hot spots results in an activation of PI3K pathway. As in other studies, these patients were considered to have a mutated gene in the analysis [39].

PTEN is a tumor suppressor gene, and can be down-regulated or lost of expression via deletion (common in breast cancer), mutation (rare in breast cancer), or promoter DNA methylation [14,41,42]. Loss of PTEN expression results in activation of PI3K pathway leading to development of cancer [15,41]. PTEN loss is present in about one third of breast cancer patients, ranging from 15% to 48% [43-46]. Our study showed that the incidence of PTEN loss was 31.6%, which is consistent with other reported results.

Previous reports suggested that PIK3CA mutation and PTEN loss were mutually exclusive [37]. However, in 4 patients with H1047R mutations in our study, 3 patients were also found to have no PTEN expression. This fact was previously reported by Perez-Tenorio et al in 2009 [40]. PI3K mutation was indicated to be associated with ER positivity, HER2 negativity and primary tumor size, which were not observed in our study [37,39,40].

An analysis of our data showed that patients with PI3K pathway activation had a statistically significant shorter median PFS than those with no PI3K pathway activation (4.5 versus 9.0 months, P = 0.013), confirming the reported conclusion that PI3K pathway activation can result in resistance to trastuzumab [6-10]. Based on the published preclinical studies, these patients should be sensitive to lapatinib, a drug with a different mechanism of action [47]. There were some clinical data demonstrating that lapatinib induced objective responses in patients who had failed trastuzumab [22]. However, all patients were treated with lapatinib and capecitabine in our study, and PI3K pathway activation was still correlated with a lower clinical benefit rate (36.4% versus 68.6%, P = 0.017) and a lower overall response rate (9.1% versus 31.4%, P = 0.05), which is consistent with results of a smaller study reported by Cizkova et al [48]. Campone [49] et al pointed out that acquisition of resistance is frequently linked to an uncoupling between upstream signals emanating from HER2 itself and downstream signals related to PI3K, AKT and/or MAPK. Two studies showed that both knockdown of PTEN and transfection of mutant PIK3CA can result in lapatinib resistance and the mTOR/PI3K inhibitor, NVP-BEZ235 can reverse the resistance [26,50].

However, there are also a few converse opinions. Based on the experimental results, O'Brien et al showed that lapatinib could overcome trastuzumab resistance via continued deactivation of PI3K/AKT/mTOR signaling [51]. A Japanese clinical study recruiting 122 patients attempted to illustrate the relationship between PI3K pathway activation and efficacy of lapatinib, but PIK3CA mutation was only found in 3 tissue samples among all 29 analyzed samples [52]. Recently, Toi et al indicated that low PTEN could predict response to lapatinib in a small phase 2 neoadjuvant trial [53]. Therefore, a definite conclusion regarding the PI3K pathway status and anti-HER2 therapy cannot be drawn up to now, and our study justifies further research.

It remains controversial whether the two gene alterations have any prognostic value. Li et al suggested that PIK3CA mutation was a negative prognostic factor [35]. On the contrary, a larger sample size study and a Japanese study indicated that it was a positive prognostic factor [36,39]. Barbareschi [54] et al reported that mutation in exon 20 usually indicated good prognosis, while the mutation in exon 9 often meant bad prognosis. Perez-Tenorio [40] et al suggested that the two gene alterations should be combined with S phase fraction to give an accurate prediction of prognosis. Recently, Dupont Jensen [55] et al showed that there is a discrepancy of PIK3CA mutation between primary and metastatic tumors, urging on a simultaneous detection of the two matched samples. For the prognostic value of PTEN, it is relatively uniform and most investigators thought that PTEN loss is a negative prognostic factor [56,57]. Our data showed that it was statistically associated with clinical benefit rate (P = 0.021). Due to a relatively smaller sample size of our study, no significant correlations between PI3K pathway status and clinicopathological parameters were found.

## Conclusions

In conclusion, PIK3CA mutation occurs more frequently in elder patients and the ratio of mutations in hot spots to non-hot spots is about 2.5 to 1 in HER2-positive breast cancer patients. PTEN loss is present in about one third of patients. PIK3CA mutation and PTEN loss were not mutually exclusive. PI3K pathway activation may lead to drug resistance to lapatinib as well as trastuzumab.

## Abbreviations

PTEN: Phosphatase and tensin homolog deleted on chromosome ten; PI3K: Phosphatidylinositol-3-kinase; PIK3CA: Phosphatidylinositol-3-kinase catalytic subunit; EGFR: Epidermal Growth Factor Receptor; HER2: Human Epidermal Growth Factor Receptor 2; PFS: Progression Free Survival; OS: Overall Survival; ORR: Overall Response Rate; CBR: Clinical Benefit Rate.

## Competing interests

The authors declare that they have no competing interests.

## Authors' contributions

LW designed the study, carried out the experiments, statistical analysis and drafted the manuscript. QZ contributed equally. JZ, SS, HG, ZJ, BW, ZS and ZW participated in the clinical work. XH proposed this study, organized the research team, interpreted all the data, and writing the manuscript. All authors read and approved the final manuscript.

## Pre-publication history

The pre-publication history for this paper can be accessed here:

http://www.biomedcentral.com/1471-2407/11/248/prepub
